# Eliciting Survival Expectations of the Elderly in Low-Income Countries: Evidence From India

**DOI:** 10.1007/s13524-017-0560-8

**Published:** 2017-03-09

**Authors:** Adeline Delavande, Jinkook Lee, Seetha Menon

**Affiliations:** 1 Institute for Social and Economic Research (ISER), University of Essex, Colchester, UK; 2 Nova School of Business and Economics, Lisbon, Portugal; 3 University of Southern California, Los Angeles, CA USA; 4 RAND Corporation, Santa Monica, CA USA; 5 Max Weber Fellow, Department of Economics, European University Institute, Fiesole, Italy

**Keywords:** Survival expectations, Aging, Biomarkers

## Abstract

We examine several methodological considerations when eliciting probabilistic expectations in a developing country context using the Longitudinal Ageing Study in India (LASI). We conclude that although, on average, individuals are able to understand the concept of probability, responses are sensitive to framing effects and to own versus hypothetical-person effects. We find that overall, people are pessimistic about their survival probabilities compared with state-specific life tables and that socioeconomic status does influence beliefs about own survival expectations as found in previous literature in other countries. Higher levels of education and income have a positive association with survival expectations, and these associations persist even when conditioning on self-reported health. The results remain robust to several alternative specifications. We then compare the survival measures with objective measures of health. We find that activities of daily life, height, and low hemoglobin levels covary with subjective expectations in expected directions.

**Electronic supplementary material:**

The online version of this article (doi:10.1007/s13524-017-0560-8) contains supplementary material, which is available to authorized users.

## Introduction

India, with 1.27 billion inhabitants, has a growing elderly population. As of this writing, 60 million people are aged 65 or older—a figure that is projected to climb to 222 million by 2050 (Central Intelligence Agency 2010; United Nations 2010). An Indian born in 1950 could expect to live for 37 years; comparatively, an Indian born today can expect to live for 69 years. This dramatic increase in the elderly dependency ratio, presents serious impending economic and health challenges that are of particular concern given the level of unemployment and poverty in the country as well as the lack of an effective health care or pension system. This increase in life expectancy also has potentially important ramifications for the many intertemporal decisions (such as retirement, bequest, investment, saving, migration, and health care) that elderly individuals have to make.

In order to make such decisions, individuals are thought to form subjective expectations about their survival. Preferences (e.g., over future consumption) and expectations (e.g., about survival) are then combined to reach a choice (e.g., current consumption and saving for the future) within existing constraints (e.g., wealth and earnings) (Hamermesh 1985; Hurd 2009). Until recently, the standard practice in economics has been to assume that individuals have rational expectations and to use population life tables in lieu of the subjective probabilities of survival. These assumptions are problematic for various reasons. First, even under rational expectations, individual-level expectations need not equal life table estimates because individuals may form their expectations based on a richer information set than that captured by life tables. Second, individuals may not have rational expectations. These assumptions can be relaxed by asking survey respondents about their expectations directly. In this article, we present unique new evidence on the subjective survival expectations of older Indians.

Asking respondents about verbal expectations—for example, “Is this event very likely or very unlikely?”—is a common practice in surveys, but responses to these questions yield only ordinal measures of beliefs. Moreover, responses may not be interpersonally comparable. These concerns lead to the elicitation of *probabilistic expectations*, wherein respondents are asked a question that can be interpreted as a probability. Manski (2004) and Hurd (2009) reviewed the literature on the elicitation of probabilistic expectations in developed countries, and Delavande et al. (2011b) and Delavande (2014) reviewed the parallel literature in developing countries. Both strands of literature emphasize that (1) survey respondents are able and willing to provide their expectations in probabilistic format, (2) a majority of respondents respect basic properties of probabilities, (3) substantial heterogeneity in beliefs exists, (4) expectations tend to vary with observable characteristics in the same way as actual outcomes, and (5) expectations are useful predictors of future behavior. However, the literature in developing countries has typically focused on younger respondents, and little is known about whether these findings apply to older individuals. We present findings from a new module designed by two of the authors and fielded in the Longitudinal Ageing Study in India (LASI).

In developed country surveys, the standard method of eliciting subjective probabilities relies on a percentage chance format: for example, “What is the percent chance that you will live to be 75 or more?,” as in the U.S. Health and Retirement Study (HRS). However, this method may be challenging in low-numeracy contexts. The most common survey approach in developing countries has been to use visual aids (such as stones, beans, or marbles) to help respondents to express probabilities. For example, Delavande and Kohler (2009), in the Malawi Longitudinal Study of Families and Health (MLSFH), asked respondents to use up to 10 beans to express the likelihood of an event happening. The data from our study use a similar approach: using up to 10 beans, respondents are asked to express the likelihood of being alive in 1 year, 5 years, and 10 years.

Since little is known about the best way to elicit survival expectations from the elderly in developing countries, our design explicitly addresses several methodological considerations to provide information on the best way to collect these expectations and to assess their validity and usefulness. First, we ask respondents both about their own survival and about the survival of people like themselves, given that respondents may be reluctant to think about their own demise. Second, we randomize the wording in terms of mortality or survival to assess any potential framing effect. In order to assess validity, we investigate how the elicited expectations relate to socioeconomic characteristics and to health biomarkers collected as part of the survey. To our knowledge, ours is the first study to assess the predictive power of biomarkers on survival expectations. Finally, we evaluate the relationship between the survival expectations and some intertemporal economic decisions.

We present results from a relatively large-scale sample representative of India’s demographic, economic, health, and cultural diversity.^1^ Our findings show great promise to elicit subjective expectations from elderly in a context such as India. First, response rates are high (e.g., approximately 87 % for one’s own probability of survival). Second, violation of the monotonicity property of probabilities is similar among older Indians and older Americans. Third, as one would expect, average survival expectations decrease as the time horizon considered increases. Fourth, survival expectations vary with observable characteristics as envisaged: younger respondents, as well as those with more education, from higher caste, with better self-reported health, and with fewer difficulties in their activities of daily living report higher survival expectations on average. Fifth, shorter respondents (an indicator of poor childhood nutrition; Steckel 1979) and men with decreased hemoglobin concentration (an indicator of anemia) report lower survival expectations, on average. Finally, respondents who have a higher 1-year survival expectation are more likely to have an outstanding loan, consistent with the idea that they are making an investment for the future. However, we also find that respondents between ages 45 and 64 are much more pessimistic about their survival than warranted by existing life table estimates—a pattern seen in other contexts (e.g., Malawi or the United States). Women also appear more pessimistic than men, which is also a pattern that has been seen in other contexts (Malawi, United States, Europe).

From a methodological point of view, our findings offer some insights into the best way to elicit subjective expectations from older respondents in a context such as India. First, response rates are not significantly improved by asking about the survival of “people like you” instead of own survival. However, answers can differ greatly depending on respondents’ perceptions of own health. Researchers interested in learning about respondents’ own survival should therefore ask about it directly. Second, framing the question in terms of survival or mortality influences respondents’ answers. The mean and median subjective survival probability when asked the survival format is higher than when asked the mortality format for both own survival and hypothetical-person survival. For the longer time horizons, the difference is quite substantial: for example, 11 percentage points for the 10-year own survival. After we control for other covariates, this framing effect is observed for the 10-year time horizon only, when uncertainty is likely to be larger, suggesting that responses to expectation questions are reasonable.

In this article, we complement the existing literature investigating individuals’ survival expectations. Very few studies have investigated subjective survival expectations in developing countries. Delavande and Kohler (2009) looked at survival expectations in Malawi. Like in India, the reported subjective expectations about mortality correspond in broad terms with the actual variation in mortality—for example, respondents living in regions with higher mortality risks have higher mortality expectations—but they are widely overestimated. Aguila et al. (2014), reporting the results of various cognitive interviews to assess the best way to elicit survival expectations from older Mexicans, emphasized the usefulness of visual aids. In the U.S. context, a number of in-depth studies have been conducted using the subjective expectations from the HRS. They appear well calibrated, on average; vary systematically with known risk factors; and evolve in panel in response to information relevant to survival, such as parental death or onset of disease. For instance, Hurd and McGarry (1995) showed that survival expectations are internally consistent and are good approximations to population probabilities. Schoenbaum (1997) compared the subjective survival expectations of smokers to smoking-specific life tables from nationally representative data on the United States and found that survival expectations were close to actuarial predictions. Subjective survival expectations have also been found to be predictive of actual survival (Bloom et al. 2006; Delavande and Rohwedder 2011a; Elder 2007; Hurd and McGarry 2002; Perozek 2008). Similar findings have been reported based on subjective probabilities of survival elicited in the English Longitudinal Study of Ageing (ELSA) and the Survey of Health, Ageing and Retirement in Europe (SHARE) (e.g., Balia 2014; Delavande and Rohwedder 2011a; Hurd et al. 2004; Menon 2015; Winter 2008).

## Data Description

### Longitudinal Ageing Study in India (LASI)

We use data collected in the LASI pilot survey, which was fielded between October and December, 2010. LASI collected data on health, retirement, and economic and social well-being of India’s elderly population. LASI consists of a household survey, collected once per household, and an individual survey for each age-eligible respondent (at least 45 years of age) and the respondent’s spouse. The LASI instrument was developed to be internationally comparable with the HRS of the United States and is harmonized to other surveys such as the China Health and Retirement Longitudinal Study (CHARLS). To capture India’s demographic, economic, health, and cultural diversity, the LASI pilot selected two northern states (Punjab and Rajasthan) and two southern states (Karnataka and Kerala). A representative sample from these four states was drawn, using a stratified, multistage, area probability sampling strategy. From each state, two districts were selected at random from the 2001 census districts and eight primary sampling units (PSUs) randomly from each district. PSUs were chosen to match the urban/rural share of the population, and 25 residential households were then selected through random sampling from each PSU, from which an average of 16 households contained at least one age-eligible individual (Arokiasamy et al. 2012). The LASI pilot achieved an individual response rate of 90.9 %. The total individual sample size is 1,683 respondents within 950 households, of whom 1,486 are aged 45 years or older.

### Expectations Module

LASI implemented an expectations module to a randomly selected 33 % of the total number of respondents. This module included questions about subjective probabilities of survival to specific ages. Respondents were given preliminary training questions to introduce them to the concept of probability. Of the 1,486 age-eligible respondents, 531 were asked the expectations module. Of these, 467 respondents are 45 years or older, which is our analytical sample. The expectations module took an average of five minutes to complete. The module uses an interactive elicitation technique based on asking respondents to allocate up to 10 beans on a plate to express the likelihood that an event will be realized (Delavande and Kohler 2009). Using 10 beans forced respondents to round their answers to the nearest 10 % but was chosen to reduce the cognitive burden on respondents (see discussion in Delavande et al. 2011b). Prior to eliciting subjective survival probabilities, the respondents were given an explanation of basic probability concepts and given the following introduction:

I will ask you several questions about the chance or likelihood that certain events are going to happen. There are 10 beans in the cup. I would like you to choose some beans out of these 10 beans and put them in the plate to help me understand what you think the likelihood or chance is of a specific event happening. If you do not put any beans in the plate, it means you are sure that the event will NOT happen. If you add beans, this means that you think the likelihood that the event happens will increase. For example, if you put in one or two beans, it means you think the event is not likely to happen but it is still possible. If you pick 5 beans, it means that it is just as likely it happens as it does not happen (fifty-fifty). If you pick 6 beans, it means the event is slightly more likely to happen than not to happen. If you put 10 beans in the plate, it means you are sure the event will happen. One bean represents one chance out of 10. There is not a right or wrong answer; I just want to know what you think.

Our analysis focuses on survival expectations. Respondents were asked about their survival in 1 year, 5 years, and 10 years. Two important features of the design have methodological relevance. First, the wording of the questions in terms of survival (alive) or mortality (not alive) was randomized. Second, all respondents were asked for both their own survival expectations and survival expectations of a hypothetical individual like themselves. Of the 467 age-eligible respondents who answered the expectations module, 239 were asked mortality questions, and 228 were asked survival questions. The questions were organized and worded as follows:

1. Mortality wording

I would like to ask you to consider the likelihood that you and other people may not be alive as time goes by. Think about 10 people like you (same age, gender, income, etc.).

Pick the number of beans that reflects how many

- Will die within a 1-year period beginning today.
- Will die within a 5-year period beginning today.
- Will die within a 10-year period beginning today.

Now, I would like to ask you to consider the likelihood that you may not be alive as time goes by. We hope that nothing bad will happen to you, but nevertheless, something unfortunate may occur over the next years despite all precautions that you may take. If you don’t want to, you do not need to answer this question. Pick the number of beans that reflects how likely you think it is that

- You will die within a 1-year period beginning today.
- You will die within a 5-year period beginning today.
- You will die within a 10-year period beginning today.

 2. Survival wording

I would like to ask you to consider the likelihood that you and other people may be alive as time goes by. Think about 10 people like you (same age, gender, income, etc.). Pick the number of beans that reflects how many

- Will be alive in 1 year.
- Will be alive in 5 years.
- Will be alive in 10 years.

Now, I would like to ask you to consider the likelihood that you may be alive as time goes by. We hope that nothing bad will happen to you, but nevertheless, something unfortunate may occur over the next years despite all precautions that you may take. If you don’t want to, you do not need to answer this question. Pick the number of beans that reflects how likely you think it is that

- You will be alive in 1 year.
- You will be alive in 5 years.
- You will be alive in 10 years.

### Demographic Characteristics of the Analytical Sample

Of the 467 age-eligible respondents who were asked the expectations module, 391 respondents have full information on all demographic variables of interest. Our analysis using demographic controls is thus restricted to these 391 respondents to ensure that results are not driven by differing sample compositions. Table 1 presents the demographic composition of this analytical sample: respondents who were selected to answer the expectation module. Male respondents and female respondents are almost equally represented in the sample. In our sample, 46 % are 45–54 years of age; 8 % are older than 75 years, with the oldest respondent being 96. In addition, 37 % of the analytical sample belongs to the high/other caste community, with the rest being divided into each of the three lower caste communities. Nearly one-half (46 %) of the sample has no schooling. There are gender differences in educational attainment, with males having overall greater educational attainment than females, which is consistent with the gender differences in the national representation of educational attainment. The income variable used is self-rated by the respondent in answer to the question, “Compared to other households in this (geographic) community, how do you consider your household?” The responses were recorded in five income groups. The top two groups of *well off* and *very well off* have been collapsed because of small numbers in these categories. There is an almost equal representation from each of the four surveyed states. For 7 % of the respondents, both parents were alive at the time of the survey. More than one-half (62 %) of the respondents reported their health as being good or very good.

**Table 1:** Summary statistics of demographics

Variable	*N*	Mean
Gender		
Male	391	0.50
Female	391	0.50
Age		
45–54	391	0.46
55–64	391	0.29
65–74	391	0.17
75+	391	0.08
Caste		
Scheduled caste	391	0.14
Scheduled tribe	391	0.12
Other backward class	391	0.37
Other caste	391	0.37
Education		
No schooling	391	0.46
Primary/middle school	391	0.36
High school or more	391	0.18
Income		
Well below average	391	0.16
Below average	391	0.29
About average	391	0.47
Well off	391	0.08
State		
Punjab	391	0.26
Rajasthan	391	0.24
Kerala	391	0.25
Karnataka	391	0.25
Survival Format	391	0.50
Either Parent Is Dead	391	0.93
Self-reported Health		
Very good	391	0.02
Good	391	0.60
Fair	391	0.31
Poor	391	0.06
Very poor	391	0.02
Objective Measures of Health		
Activity of daily life	319	0.00
Height	370	159.08
High blood pressure	309	0.19
Anemia	343	0.19
Financial Variables		
Savings (INR)	129	35,520.54
Outstanding bank loan	391	0.13
Activities of Daily Living Dimensions		
Difficulty with dressing	391	0.06
Difficulty with walking	391	0.07
Difficulty with bathing or showering	391	0.04
Difficulty eating	391	0.05
Difficulty getting in or out of bed	391	0.08
Difficulty using toilet	391	0.05

*Notes:* Survival format is a binary indicator, where 1 is survival framing of the survival expectations questions, and 0 is the mortality framing of the survival expectations questions. Height is measured in centimeters. Savings is measured in Indian rupees. All other covariates are binary indicators.

## Can We Ask Survival Expectations of Older Respondents in Low-Income Countries? Methodological Considerations

In this section, we review the methodological considerations to be taken into account when eliciting survival probabilities from an older population in a developing country. We use the age-eligible sample of 467 respondents for this section to take advantage of the larger sample size and to enable reporting of response rates. Note that respondents were willing to report their beliefs in probabilistic formats: response rates are high for own probability of survival, about 87 %.

### Do Older Respondents Understand the Concept of Probabilities?

After reading the introduction, the interviewers checked whether the respondents understood the concepts of probability with some practice questions.

Respondents were then asked to pick the number of beans that reflects the probability of going to the market within two days and within two weeks to assess whether they would respect the monotonicity property of nested events. Of the 467 respondents, 447 answered this question, which translated to a 4 % nonresponse rate. Figure S1 in Online Resource 1 presents the difference in the probability of going to the market within two days and the probability of going to the market within two weeks for 447 of the respondents for whom we have complete data. A negative statistic is a violation of the monotonicity criterion of nested events, occurring in 21 % of the sample, which is consistent with previous studies eliciting subjective expectations among the elderly in developed countries (approximately 23 % in the survival expectations questions in the HRS). Among those who violated monotonicity, 54 % had no schooling—a higher percentage than reported in other developing countries with a younger sample. For example, Delavande and Kohler (2009) found that 1.41 % of their sample in Malawi violated monotonicity when asked the probability of going to the market in the first instance.

The respondents who violated the criterion were subsequently given the following information:

Remember, as time goes by, you may find more time to go to the market. Therefore, there is a higher chance that you go to the market within 2 weeks than within 2 days. So you should put more beans for the likelihood of going to the market within 2 weeks than within 2 days. Let me ask you again.

These respondents were then asked the question regarding the probability of going to the market again. Only 20 of the 447 respondents continued to violate the criterion.

Respondents were also asked a question to assess whether they understood that complementary events have a probability summing to 1. In particular, in the context of a game of Ludo, the question was as follows:

Pick the number of beans that reflects how likely you think it is that

- You will win the game.
- You will lose the game.

Of the 467 respondents, 423 answered this question, and 35 % of this sample correctly assigned probabilities to each outcome so that the sum of both would be equal to 1. Because respondents were given 10 beans, with each bean representing a 10 % likelihood, it is plausible that respondents were rounding their actual probabilities (Manski and Molinari 2010). The sum of the proportion of the sample that reported probabilities between +1 and –1 is 52 %. We are not aware of other surveys asking similar questions about complementary events, so we do not have a benchmark. Overall, respondents seem more familiar with the idea of monotonicity than complementarity.

### Survival Expectations by Time Horizon

**Fig. 1:**
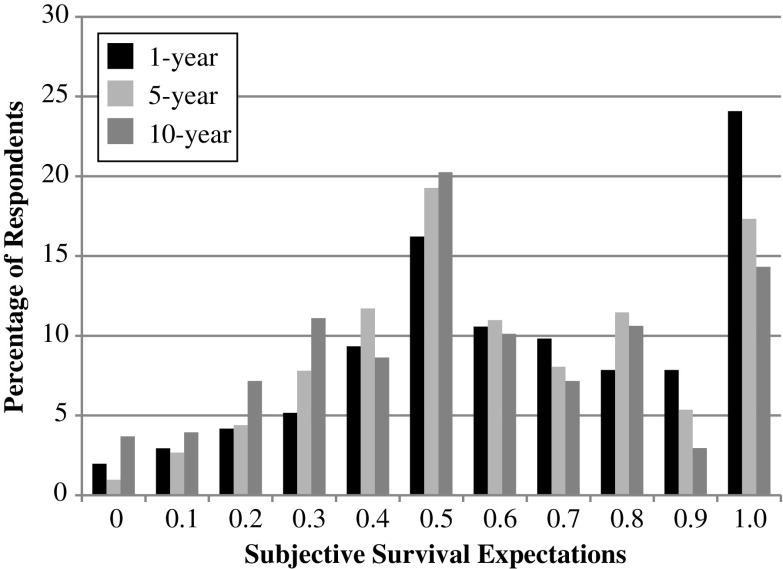
Distribution of subjective probability of survival (weighted)

To compare the various formats used to elicit expectations, we recode the mortality responses into survival and express all responses in survival terms on a scale from 0 to 1. Figure 1 shows the distribution of all three survival periods. The figure shows that respondents are aware that survival probability decreases as the time horizon increases. For example, the percentage of respondents who report a survival probability of 1 in the 1-year period is 24 % compared with 14 % of respondents who reported the same for the 10-year survival period. When looking at monotonicity violations in the survival expectations responses at the individual level, we find that (on average) 25 % of the respondents violate monotonicity for all three periods. Specifically, when comparing the 1-year survival period with the 5-year survival period, 26 % of respondents violate monotonicity. When comparing the 5-year with the 10-year survival period and the 1-year with the 10-year survival period, monotonicity violations are at 22 % and 27 %, respectively.

Heaping at 0.5 is also a common feature of subjective expectations. Previous studies have shown that expectations of 0.5 may be indicative of epistemic uncertainty (e.g., de Bruin et al. 2000). This previous finding is consistent in the LASI data with uncertainty increasing as the time horizon increases. Respondents are more likely to report 0.5 in the 5-year and 10-year survival periods of 19.27 % and 20.25 %, respectively, compared with 16.22 % in the 1-year survival period.

### You Versus Other People Like You

In addition to being asked to report their own survival expectations, respondents were asked to think of 10 people like themselves and to report the survival expectations for these hypothetical individuals. This has been done in previous studies (e.g., Aguila et al. 2014; McKenzie et al. 2008). The potential advantage of this question wording is to improve response rates, given that people may be less reluctant to think about the mortality of others. However, it is conceptually a different expectation than one’s own expectations: answers may vary between own and hypothetical-person survival because respondents may make unobservable assumptions about the characteristics of the hypothetical individuals. Researchers interested in explaining how mortality expectations influence *individual* decision-making want to elicit respondents’ own expectations. There may be trade-off between better response rates and precise survival estimates (see discussion in Delavande 2014). Panel A in Table 2 presents the summary statistics of own survival and hypothetical-person survival. Response rates are only slightly higher for hypothetical person’s survival probability than for own survival probability and are not statistically significantly different. Regarding the average levels of expectations, beliefs about own survival relative to a hypothetical person’s survival are similar. The unpaired *t* test for equality in means between own survival and the survival of a hypothetical person is not significant in any of the three time frames.

**Table 2:** Summary statistics of subjective survival expectations

Statistics	Own Mortality			Hypothetical-Person Mortality		
	1 Years	5 Years	10 Years	1 Years	5 Years	10 Years
A. Summary Statistics of Own- Versus Hypothetical-Person Survival						
Mean	.65	.61	.55	.63	.61	.56
*p*25	.60	.60	.50	.60	.60	.50
*p*50	.50	.40	.30	.50	.40	.40
*p*75	.90	.80	.80	.90	.80	.80
*p* values of unpaired *t* test for equality of means^a^				.32	.89	.73
*N*	407	410	405	423	420	420
Response rate	.87	.88	.87	.91	.90	.90
*p* values of unpaired *t* test for equality of response rates^b^				.10	.30	.13
B. Summary Statistics of the Difference Between Own Survival and Hypothetical-Person Survival						
Percentage with different responses				53.96	55.03	55.89
Mean				.03	.01	–.01
*p*25				.10	.10	.10
*p*50				–.20	–.20	–.20
*p*75				.20	.20	.20
*p* values of unpaired *t* test for mean different from zero				.91	.90	.38
*N*				2.52	2.57	2.61

^a^Unpaired *t* test for equality of means between own versus hypothetical survival.

^b^Unpaired *t* test for equality of response rates between own versus hypothetical survival.

We further investigate the difference of expectations at the individual levels. Panel B in Table 2 presents the summary statistics of the difference in the responses between own survival and hypothetical-person survival for those respondents who provided different answers. Approximately 55 % of the respondents reported a different answer. The differences in the responses, on average, are quite small, varying between −0.01 and 0.02 in the three survival periods. However, the percentiles show that the differences can be large for some individuals. For example, the 25th and 75th percentiles correspond to a very large difference of 20 percentage points.

In Table S1 in Online Resource 1, we seek to evaluate whether individual characteristics and self-reported health are predictive of the difference between own survival and that of the hypothetical individual. For this analysis, we restrict the sample to respondents whose responses differed between the own survival and hypothetical-person survival wording of the questionnaire. Table S1 presents the ordinary least squares (OLS) coefficients using the difference in beliefs as dependent variables. Demographic and socioeconomic characteristics have essentially no predictive power for this difference. However, as one would expect, respondents with relatively poor self-reported health status were also likely to report differential survival probabilities compared with a hypothetical individual.

Overall, in the context of this study, response rates are not significantly improved by asking about the survival of “people like you” instead of own survival. However, there can be large differences in answers, driven by the perception of own health. Researchers interested in learning about respondents’ own survival should ask about it directly.

### Mortality Versus Survival

Previous studies have shown that framing can have an effect on survey responses (e.g., Tversky and Kahneman 1981). Studies examining the framing effect specifically on survival and mortality format of questionnaires have shown mixed results. Some studies have failed to find a significant effect (e.g., Miller and Fagley 1991), whereas some studies have found a significant effect that is small in magnitude (for an overview, see Levin et al. 1998).

Respondents in the expectations module of LASI were randomized between the survival format of the question and the mortality format of the question (see the earlier section, Expectations Module). Table 3 presents the summary statistics for the mortality versus survival question format. To enable comparison, we recode responses to the mortality format of the questionnaire in survival terms. The question format does not seem to systematically influence response rate: the difference in response rate is not statistically different across the two formats. However, respondents reported feeling a little uncomfortable talking about their own mortality to interviewers.

**Table 3:** Summary statistics of mortality format versus survival format

Statistics	Own Mortality			Hypothetical-Person Mortality		
	1 Years	5 Years	10 Years	1 Years	5 Years	10 Years
Mortality Format						
Mean	.64	.58	.50	.62	.60	.51
*p*25	.60	.50	.50	.60	.50	.50
*p*50	.40	.40	.30	.40	.40	.40
*p*75	.90	.80	.70	.90	.80	.70
*N*	205	203	203	214	214	213
Response rate	.86	.85	.85	.90	.90	.89
Survival Format						
Mean	.67	.65	.61	.65	.62	.61
*p*25	.70	.70	.60	.60	.60	.60
*p*50	.50	.50	.50	.50	.50	.50
*p*75	.10	.90	.80	.90	.80	.80
*N*	202	207	202	209	206	207
Response rate	.89	.91	.89	.92	.90	.91
*p* Values of Unpaired *t* Test for Equality of Means^a^	.21	.01	.00	.35	.34	.00
*p* Values of Unpaired *t* Test for Equality of Response Rates^b^	.84	.79	.95	.74	.60	.69

^a^Unpaired *t* test for equality of means between mortality and survival format of the questionnaire.

^b^Unpaired *t* test for equality of response rates between mortality and survival format of the questionnaire.

The mean and median subjective survival probability when asked the survival format is higher than when asked the mortality format for both own survival and hypothetical-person survival. For the larger time horizons, the difference is substantial: for example, 11 percentage points for the 10-year own survival. The *t* test for equality of means between mortality and survival format of the questionnaire is significant at 5 % in the 5-year and 10-year survival period for own survival and in the 10-year survival period for the survival of a hypothetical individual. Therefore, a framing effect is evident for longer time horizons, with respondents allocated in the mortality format being more pessimistic about survival than those allocated in the survival format. After we control for other covariates, the framing effect is observed for the 10-year time horizon only, when uncertainty is likely to be larger (see upcoming discussion in the section, Self-reported Health).

### Do Subjective Probabilities of Survival Vary by Socioeconomic Characteristics?

We now investigate whether the subjective probability of survival varies with socioeconomic characteristics similarly given that actual survival is known to vary with those. Table 4 presents the mean subjective probability of own survival and hypothetical-person survival by characteristics. Means are weighted by the pooled individual weight to provide survey design–adjusted standard errors across the four states.

**Table 4:** Mean subjective probability of survival (weighted)

	Own Survival			Hypothetical-Person Survival		
	1-Year Survival	5-Year Survival	10-Year Survival	1-Year Survival	5-Year Survival	10-Year Survival
All	.65	.61	.55	.63	.61	.56
Men	.65	.62	.56	.63	.62	.58
Women	.65	.61	.55	.64	.60	.55
Age						
45–54	.65	.63	.57	.63	.63	.58
55–64	.69	.64	.58	.67	.62	.56
65–74	.64	.59	.48	.60	.56	.53
75+	.60	.51	.48	.55	.57	.50
Caste						
Scheduled caste	.60	.59	.53	.60	.59	.51
Scheduled tribe	.59	.59	.56	.61	.59	.59
Other backward class	.66	.61	.55	.62	.62	.58
Other caste	.68	.63	.56	.66	.62	.55
Education						
No schooling	.60	.58	.54	.58	.58	.55
Primary/middle schooling	.68	.62	.53	.67	.64	.54
High school or more	.73	.70	.63	.69	.65	.61
Income						
Well below average	.57	.60	.59	.60	.59	.58
Below average	.69	.63	.56	.63	.63	.56
About average	.67	.61	.53	.65	.61	.55
Well off	.56	.57	.54	.62	.59	.55
State						
Punjab	.63	.58	.52	.60	.57	.48
Rajasthan	.67	.65	.61	.65	.63	.64
Kerala	.80	.71	.58	.76	.67	.57
Karnataka	.51	.52	.51	.52	.57	.55
*N*	407	410	405	423	420	420

*Notes:* Means are weighted by the pooled individual weights to provide survey design–adjusted standard errors. All variables are coded as binary indicators.

We offer a few important remarks based on this table. First, as already shown in the Expectations Module section, survival expectations decrease as the time horizon considered increases: for example, the difference in survival subjective probability within 1 year and within 10 years is 10 percentage points. Second, in almost all cases, survival expectations decrease as age increases. For example, respondents aged 45 to 54 expect a 63 % chance of being alive in the next 5 years, on average; those aged 75+ expect a 51 % chance. Third, a clear caste and education gradient is evident in the responses. High caste respondents report higher expectations of own survival in all three time horizons. Respondents with at least a high school education report higher survival expectations in all three time horizons for both own survival and the survival of a hypothetical person. Fourth, women and men have similar levels of expectations, although women have greater life expectancy (male life expectancy at birth is 63 years, and female life expectancy at birth is 66 years; World Health Organization 2011). This female pessimism has previously been documented in other contexts such as Malawi, several European countries, and the United States (e.g., Delavande and Kohler 2009; Delavande and Rohwedder 2011b; Dormont et al. 2014; Hurd 2009).

The survival responses according to the income category of the respondent are mixed. Respondents from households with income well below average reported lower survival probabilities, as did the most affluent respondents. A possible explanation is that respondents with very high income may be more health literate and so may adjust their survival expectations accordingly (Bloom 2005). Considerable heterogeneity exists between states, with Karnataka reporting lower survival responses than the other states in all three periods and for own and hypothetical-person survival.

### Assessing Accuracy of Survival Expectations

To further assess the validity of respondents’ survival expectations, we compare subjective survival expectations to life table estimates. Even under rational expectations, individual-level expectations need not equal life table estimates because individuals may form their expectations based on a richer information set (e.g., health behavior, parental survival, knowledge of chronic disease) than the one captured by life tables, which typically condition on age and gender. However, they should match in the aggregate. If respondents have accurate beliefs about their survival, the average of the subjective probabilities would be close to the expected value of actual survival (e.g., Hamermesh 1985; Hurd 2009). We use life table estimates based on the Sample Registration System (SRS) and published by the Government of India (Office of Registrar General 2012). The SRS is a large-scale demographic survey based on a dual recording system that provides reliable mortality estimates at state and national levels. Abridged life tables are created using the mortality package MORTPACK 4, the UN’s software package for mortality measurements. For the purpose of our analysis, we use the revised life table reports for the period 2006–2010. The comparison is therefore not completely ideal to assess accuracy given that we are comparing 2010 life tables with prospective survival, but it is still a useful exercise.

Panel A in Table 5 presents the state life table estimates for the 5-year and 10-year survival periods for the overall sample and the state-specific life table estimates. Panel B presents the overall and state-wise subjective survival estimates.

**Table 5:** State life table summary statistics

Statistics	Overall		Punjab		Rajasthan		Kerala		Karnataka	
	5-Year Survival	10-Year Survival	5-Year Survival	10-Year Survival	5-Year Survival	10-Year Survival	5-Year Survival	10-Year Survival	5- Year Survival	10-Year Survival
A. State Life Table Estimates										
Mean	.84	.68	.82	.66	.82	.66	.83	.66	.88	.73
*p*50	.91	.78	.89	.74	.92	.81	.88	.73	.92	.80
*p*25	.79	.56	.79	.55	.73	.47	.79	.56	.87	.69
*p*75	.94	.86	.93	.84	.95	.87	.94	.85	.95	.87
*N*	400	400	103	103	95	95	98	98	104	104
B. Subjective Survival Expectations										
Mean	.61	.56	.58	.52	.65	.62	.71	.59	.52	.51
*p*50	.60	.50	.60	.50	.60	.60	.80	.55	.50	.50
*p*25	.40	.30	.40	.30	.40	.40	.50	.40	.40	.35
*p*75	.80	.80	.80	.70	.90	.80	1.00	.80	.60	.60
*N*	400	400	103	103	95	95	98	98	104	104
*p* Value of *t* Test^a^	.00	.00	.00	.00	.00	.34	.00	.05	.00	.00

^a^Unpaired *t* test for equality of means between state life table estimates and subjective survival expectations.

Table 5 shows that people are on average much more pessimistic about their survival probabilities than is warranted by existing life table estimates. Overall, respondents reported a 61 % chance of being alive in the next five years, while the equivalent life table statistic is 84 %. Table S2 in Online Resource 1 indicates that this pessimism is driven mostly by younger respondents (aged 64 or younger). Respondents aged 65–74 similarly underestimated their 5-year survival, although they had relatively accurate 10-year survival expectations. On the contrary, respondents aged 75 or older overestimated their chance of survival. If anything, one would expect life expectancy not to deteriorate in the coming years, so this pessimism of the younger respondents is unlikely to have been driven by them being forward-looking and predicting a reduction in life expectancy. A similar age-related bias has been observed in developed countries (Hudomiet et al. 2015). The literature has offered various explanations for this bias, such as measurement error bounded by 0 % and 100 %, bias toward 50 %, uncertainty, or ambiguity (Groneck et al. 2016; Hudomiet et al. 2015, 2016).^2^

Another revelation from Table 5 is that individuals seem unaware of the protective effect of residing in certain states. In the state life table estimates, the survival forecasts are clearly ordered (i.e., Karnataka, Kerala, Punjab, and Rajasthan), reflecting a decreasing survival forecast. For both the 5-year and 10-year survival periods, Karnataka has the highest survival probabilities as reported by the state life table estimates, and Rajasthan has the lowest survival probabilities. With respect to subjective survival probabilities, such a clear ranking does not exist. Kerala has the highest survival expectation in the 5-year period, and Rajasthan has the highest in the 10-year period. Karnataka has the lowest survival expectation in both the 5-year and the 10-year periods.

One may wonder whether the subjective survival of the hypothetical individuals more closely match population life tables. We do not find that this is the case: regional averages of own survival and hypothetical individual survivals are very similar (not shown). One possible reason for this finding is that we asked respondents to assume that the hypothetical individual was like themselves (same age, gender, income, and so on). See the exact wording of the question in the earlier section, Expectations Module.

## Health Measures and Subjective Survival Expectations

In this section, we evaluate how various measures of health are correlated with the elicited survival expectations. We focus on self-reported health, activities of daily living (ADLs), and objective biomarkers. We present the best linear predictors, in line with the common practice of other studies examining the correlation of subjective probabilities on characteristics. As a robustness check, we also run regressions using the fractional logit model developed by Papke and Wooldridge (1996) for dependent variables between 0 and 1 and reassuringly find similar qualitative results (not shown).^3^^,^ 4 Note also that our estimates cannot be interpreted as causal because our measures of health and the subjective survival expectations may be correlated through unobserved variables (such as health behaviors).

### Self-reported Health

Previous studies have found that self-reported health is a good predictor of mortality (Burström and Fredlund 2001; Idler and Benyamini 1997). In the context of India, self-reported health measures have been shown to be reliable measures of health when estimates are conditioned on region (Chen and Mahal 2010).

Table 6 presents an OLS regression investigating the predictive power of self-reported health, after conditioning on demographic characteristics. As comparison, the first three columns show results when we control for demographic characteristics only.

**Table 6:** Basic regressions of sociodemographic characteristics and self-reported health on survival

	Basic Regression			Basic Regression + Self-rated Health		
	1-Year Survival	5-Year Survival	10-Year Survival	1-Year Survival	5-Year Survival	10-Year Survival
Male						
Female	0.020	0.011	–0.005	0.030	0.022	0.001
	(0.030)	(0.024)	(0.027)	(0.032)	(0.025)	(0.027)
45–54 Years						
55–64 Years	0.054^†^	0.024	0.011	0.070*	0.041	0.022
	(0.032)	(0.031)	(0.038)	(0.030)	(0.029)	(0.036)
65–74 Years	–0.008	–0.045	–0.091^†^	0.037	0.001	–0.061
	(0.039)	(0.046)	(0.046)	(0.038)	(0.040)	(0.042)
75+ Years	–0.035	–0.122**	–0.095^†^	0.018	–0.064	–0.057
	(0.051)	(0.043)	(0.055)	(0.045)	(0.047)	(0.061)
No Schooling						
Primary/Middle School	0.043	0.046	–0.004	0.046	0.053	0.000
	(0.041)	(0.037)	(0.041)	(0.040)	(0.036)	(0.040)
High School or More	0.086	0.103^†^	0.075	0.078	0.095^†^	0.07
	(0.053)	(0.053)	(0.055)	(0.051)	(0.050)	(0.053)
Other Caste						
Schedule Caste	–0.007	0.028	0.008	0.002	0.032	0.011
	(0.048)	(0.042)	(0.050)	(0.048)	(0.044)	(0.051)
Schedule Tribe	–0.034	–0.008	–0.031	–0.015	0.013	–0.017
	(0.070)	(0.054)	(0.055)	(0.069)	(0.056)	(0.058)
Other Backward Caste	0.017	0.008	–0.004	0.029	0.013	0.001
	(0.035)	(0.033)	(0.035)	(0.036)	(0.033)	(0.036)
Mortality Format						
Survival Format	0.028	0.057^†^	0.125**	0.019	0.047	0.119**
	(0.031)	(0.032)	(0.034)	(0.031)	(0.032)	(0.035)
Both Parents Alive						
One or Both Parents Are Dead	–0.136**	–0.102^†^	–0.048	–0.148**	–0.115*	–0.055
	(0.049)	(0.054)	(0.046)	(0.050)	(0.047)	(0.042)
Income, Well Below Average						
Income, Below Average	0.090*	0.028	–0.033	0.102*	0.035	–0.026
	(0.044)	(0.040)	(0.045)	(0.043)	(0.040)	(0.045)
Income, About Average	0.049	–0.006	–0.073^†^	0.071^†^	0.012	–0.061
	(0.041)	(0.041)	(0.041)	(0.038)	(0.043)	(0.043)
Income, Well Off	–0.019	0.002	–0.028	–0.011	0.001	–0.027
	(0.065)	(0.057)	(0.046)	(0.066)	(0.060)	(0.048)
Punjab						
Rajasthan	0.043	0.085	0.119*	0.046	0.089	0.121*
	(0.068)	(0.060)	(0.051)	(0.068)	(0.060)	(0.051)
Kerala	0.124*	0.097*	0.068	0.191**	0.152**	0.105*
	(0.050)	(0.047)	(0.045)	(0.052)	(0.050)	(0.050)
Karnataka	–0.124**	–0.080*	–0.032	–0.119**	–0.073*	–0.029
	(0.035)	(0.037)	(0.043)	(0.036)	(0.036)	(0.043)
Self-reported Health, Very Good						
Self-reported Health, Good				–0.061	–0.116	–0.081
				(0.105)	(0.108)	(0.085)
Self-reported Health, Fair				–0.149	–0.167	–0.119
				(0.107)	(0.114)	(0.096)
Self-reported Health, Poor				–0.240^†^	–0.368**	–0.214^†^
				(0.126)	(0.129)	(0.110)
Self-reported Health, Very Poor				–0.355*	–0.309*	–0.282^†^
				(0.137)	(0.137)	(0.143)
*N*	391	391	391	391	391	391

*Notes:* Regressions are weighted by the pooled individual weights to provide survey design–adjusted standard errors. Robust standard errors, clustered at state level, are shown in parentheses. 1-year survival, 5-year survival, and 10-year survival indicate the respondent’s subjective survival expectations reported for the three time frames. All covariates are coded as binary indicators.

^†^ *p* < .10; *p < .05, **p < .01

As shown in columns 4–6, self-reported health status has a negative relationship with survival probabilities, which is statistically significant in all three time horizons. The magnitude of the effect is very large: for example, those who rated their health as very poor have a subjective probability of survival that is 0.36 point lower than that for respondents who rated their health as very good. Survival expectations are therefore in line with self-reported health, even after we condition for other characteristics. As discussed earlier, some indicators of socioeconomic status and state of residence are correlated with beliefs. Also, having one or both parents dead is associated with lower probability of survival, a result commonly found in other studies (e.g., Dormont et al. 2014; Hamermesh 1985; Hurd and McGarry 1995)

Interestingly, we also find a framing effect of the questionnaire format (survival vs. mortality) in the 10-year survival period of 0.11. This finding suggests that respondents are more influenced by the framing of the questionnaire when the survival period in question is longer, and therefore when there is presumably more uncertainty.

### Activities of Daily Life

LASI also collected self-reported disability rates measured by difficulty with at least one ADL. Self-reported measures have been shown to be reliable measures of health in India (Subramanian et al. 2009). The bottom panel in Table 1 presents the proportion of respondents within the analytical sample in each measure of the ADL who reported having a difficulty. We code a factor score of ADL using the aforementioned measures through a principle component analysis. A high score on the ADL thus means the respondent does not have a difficulty in any of the six ADLs, and a low score indicates that the respondent has difficulties in one or more of the six ADLs. The top panel in Table 7 presents the estimates of the association between subjective survival probability and self-reported measures of ADL. These are based on OLS regressions similar to those in the first three columns of Table 6. Each cell in Table 7 reports the results of separate estimations with all the control variables used in the main specification. Subjective survival probabilities in the 1-year time horizon and 5-year time horizon are positively correlated with ADL measures, with coefficients of .03 and .02, respectively.

**Table 7:** Association between survival expectations and objective measures of health

	1-Year Survival	5-Year Survival	10-Year Survival
Activities of Daily Life	0.030*	0.027^†^	0.008
	(0.014)	(0.016)	(0.021)
* N*	319	318	313
High Blood Pressure	–0.041	–0.001	–0.035
	(0.038)	(0.034)	(0.038)
* N*	309	307	304
Height	0.005*	0.005*	0.005*
	(0.002)	(0.002)	(0.002)
* N*	320	319	314

*Notes:* Each cell in the above table reports the results of separate estimations with all the control variables used in columns 1–3, Table 6. Regressions are weighted by the pooled individual weights to provide survey design–adjusted standard errors. Robust standard errors, clustered at the state level, are shown in parenthesis. ADL is the first component of a principle component analysis with a mean of 0 and a standard deviation of 1, using the components shown in Table 1. High blood pressure is a binary indicator, with 1 indicating the incidence of high blood pressure. Height is measured in centimeters.

^†^ *p* < .10; *p < .05

### Biomarkers

LASI included a biomarker content, which includes anthropometric measures, blood pressure readings, vision and physical functioning test, and a collection of dried blood samples (Bloom et al. 2014). These data allow us to compare subjective survival expectations in India with objective measures of health collected through the direct assessment of biomarkers. Among the 1,683 individuals interviewed for LASI, 1,311 completed the biomarker module, which translates to a 77.9 % completion rate.

The second panel in Table 7 shows the association between high blood pressure and survival expectations. We find a negative but insignificant relationship in all three periods.

Several studies have established an association between height, early-life nutritional status, morbidity, and mortality (Bhalotra and Rawlings 2011; Monden and Smits 2009). The average height in our analytical sample is 165.5 cm for men and 153.1 cm for women. The third panel in Table 7 presents the association between height and subjective survival probability of the respondents. We see a positive relationship between height and survival probability for all three time horizons with a magnitude of .005 (*p* value < .05).

Decreased hemoglobin concentrations are an indicator for anemia, which is highly prevalent in developing countries. Lower levels of hemoglobin have been shown to predict mortality and morbidity (Guralnik et al. 2004; Tolentino and Friedman 2007). In LASI, hemoglobin levels were measured using an ELISA (enzyme-linked immunosorbent assay) protocol based on the O’Broin and Gunter (1999) method. The mean hemoglobin level for our analytical sample is 14.3 g/dl, which is slightly above the mean of the LASI biomarker sample of 14.1 g/dl. We create a binary indicator for low hemoglobin levels based on standard clinical cut points of 12.0 g/dl for women and 13.0 g/dl for men (World Health Organization 2001). Nearly one-fifth 19 % (66 respondents) of our analytical sample have low hemoglobin levels, of whom 73 % (48 respondents) are women.

We find a strong negative association between low hemoglobin concentrations and subjective survival expectations in the 1-year and 5-year period for men with magnitudes of 0.14 and 0.17, respectively, as shown in Table 8 (*p* value < .05). We find no significant effects for women.

**Table 8:** Association with low hemoglobin concentrations

	Female			Male		
	1-Year Survival	5-Year Survival	10-Year Survival	1-Year Survival	5-Year Survival	10-Year Survival
Low Hemoglobin	0.058	0.051	0.072	–0.138*	–0.167*	–0.024
	(0.036)	(0.030)	(0.050)	(0.062)	(0.068)	(0.074)
*N*	152	148	145	141	144	142

*Notes:* Each cell reports the results of separate estimations with all the control variables used in columns 1–3, Table 6. Low hemoglobin is a binary indicator, with 1 indicating hemoglobin levels below 12.0 g/dl for women and below 13.0 g/dl for men. Regressions are weighted by the pooled individual weights to provide survey design–adjusted standard errors. Robust standard errors, clustered at the state level, are shown in parentheses.

*p < .05

## Survival Expectations and Expenditure

In this section, we explore the association between survival expectations and some economic decisions of the respondents to evaluate whether survival expectations are correlated with forward-looking decisions for which how long one expects to live should matter. As argued in the introduction, an important motivation to collect expectations data is to better understand decision-making under uncertainty. Recent studies incorporating expectations into econometric models have addressed a wide range of decisions, such as contraception choice (Delavande 2008), portfolio allocation (Delavande and Rohwedder 2011b; Kézdi and Willis 2011), fertility and sexual behavior (De Paula et al. 2014; Delavande and Kohler 2016; Shapira 2013), education (Arcidiacono et al. 2012; Zafar 2013), committing a crime (Lochner 2007), migration (McKenzie et al. 2013), or the timing of Social Security claiming and retirement (Hurd et al. 2004; Van der Klaauw and Wolpin 2008). Also using survival expectations, Hurd et al. (2004) and Delavande et al. (2006) found that in the United States, people with higher subjective survival expectations claim Social Security later, effectively buying additional Social Security annuities. An important finding of this overall line of work is that heterogeneity in expectations is important to explain heterogeneity in behavior.

We use two dependent variables in our analysis: savings and loans. We expect people with higher survival expectations to be more likely to have a loan (i.e., they are making investments) and to have higher savings. Respondents were asked to provide an approximate value of savings accounts, postal accounts, and certificates of deposits.^5^ The summary statistics for these variables are provided in Table 1. We drop the top 1 % of the data (*n* = 1) to reduce the effect of outliers. The average value of savings reports was INR35,521 (Indian rupees) with a standard deviation of INR66,923. Bank loan is a binary variable, with 1 indicating that the respondent has an outstanding loan from a bank. More than one-tenth (13 %; 50 respondents) of the analytical sample reported having an outstanding bank loan. Table 9 presents the results of the association among survival expectations, outstanding loans, and savings. An increase in the 1-year survival expectation is positively associated with a 0.12 percentage point increase of having an outstanding bank loan, and the coefficient is statistically significant at 5 %. Although our estimates are not causal, this result is consistent with the idea that individuals who expect to die sooner—and thus have a shorter perceived optimization horizon—are less likely to make forward-looking investments (see discussion in Hamermesh 1985; Hurd 2009).

**Table 9:** Survival expectations, savings, and outstanding bank loans

	Loans	Loans	Loans	Savings	Savings	Savings
1-Year Survival	0.116*			–0.39		
	(0.052)			(0.356)		
5-Year Survival		–0.045			0.322	
		(0.054)			(0.361)	
10-Year Survival			–0.061			0.142
			(0.038)			(0.317)
Male						
Female	–0.021	–0.02	–0.021	0.339	0.354^†^	0.351
	(0.040)	(0.039)	(0.039)	(0.206)	(0.208)	(0.218)
45–54 Years						
55–64 Years	–0.05	–0.044	–0.045	0.607*	0.591*	0.582*
	(0.038)	(0.038)	(0.038)	(0.280)	(0.286)	(0.285)
65–74 Years	0.015	0.009	0.006	0.487^†^	0.584*	0.552*
	(0.047)	(0.047)	(0.047)	(0.256)	(0.262)	(0.256)
75+ Years	–0.046	–0.058	–0.058	0.289	0.376	0.336
	(0.058)	(0.057)	(0.058)	(0.567)	(0.542)	(0.546)
No Schooling						
Primary/Middle School	0.071*	0.079*	0.077*	0.241	0.224	0.236
	(0.035)	(0.037)	(0.037)	(0.265)	(0.263)	(0.271)
High School or More	0.031	0.046	0.045	0.757^†^	0.722^†^	0.746^†^
	(0.058)	(0.061)	(0.060)	(0.393)	(0.373)	(0.390)
Other Caste						
Schedule Caste	0.078	0.077	0.076	–1.051*	–0.990^†^	–0.981^†^
	(0.048)	(0.047)	(0.047)	(0.500)	(0.545)	(0.541)
Schedule Tribe	–0.005	–0.011	–0.012	–0.051	–0.222	–0.135
	(0.048)	(0.048)	(0.048)	(1.400)	(1.351)	(1.336)
Other Backward Caste	0.033	0.034	0.033	–0.175	–0.18	–0.161
	(0.065)	(0.066)	(0.066)	(0.403)	(0.397)	(0.402)
Income, Well Below Average						
Income, Below Average	0.081^†^	0.094*	0.091*	–1.093**	–1.174**	–1.142**
	(0.042)	(0.043)	(0.043)	(0.388)	(0.407)	(0.413)
Income, About Average	–0.012	–0.005	–0.01	–0.541^†^	–0.559^†^	–0.545^†^
	(0.043)	(0.041)	(0.042)	(0.297)	(0.289)	(0.283)
Income, Well Off	–0.028	–0.029	–0.031	–0.688	–0.713	–0.706
	(0.047)	(0.046)	(0.046)	(0.460)	(0.474)	(0.467)
Punjab						
Rajasthan	0.009	0.018	0.021	–0.383	–0.365	–0.391
	(0.039)	(0.040)	(0.039)	(0.334)	(0.341)	(0.340)
Kerala	0.342**	0.360**	0.360**	–0.071	–0.149	–0.135
	(0.059)	(0.058)	(0.059)	(0.496)	(0.473)	(0.477)
Karnataka	0.042	0.025	0.026	–1.454**	–1.350**	–1.392**
	(0.046)	(0.046)	(0.045)	(0.355)	(0.368)	(0.377)
*N*	391	391	391	129	129	129

*Notes:* Regressions are weighted by the pooled individual weights to provide survey design–adjusted standard errors. Robust standard errors, clustered at the state level, are shown in parentheses. 1-year survival, 5-year survival, and 10-year survival indicate the respondent’s subjective survival expectations reported for the three time frames between 0 and 1. Loans are a binary indicator, with 1 denoting the existence of an outstanding loan. Savings are coded in Indian rupees, with a mean of INR35,521 and a standard deviation of INR66,923. All other covariates are coded as binary indicators.

^†^ *p* < .10; *p < .05, **p < .01

We also created savings quintiles and use them as the dependent variables in the last three columns in Table 9. We find a negative association between savings and survival expectations in the 1-year period and a positive association in the 5-year and 10-year periods but with no statistical significance. Our results are not sensitive to coding savings in quartiles or deciles.

## Conclusion

This article presents a thorough investigation of older individuals’ subjective survival expectations in India. We inspect several methodological contemplations with regard to eliciting subjective survival expectations in the developing country context. We conclude that although individuals are, on average, able to understand the concept of probability, responses are sensitive to framing effects and own versus hypothetical-person effects. We also find that people younger than age 64 are pessimistic about their survival probabilities compared with state-specific life tables.

Next, we examine socioeconomic gradients in the Indian context for three periods of survival: 1-year, 5-year, and 10-year survival. We find that socioeconomic status does influence beliefs about own survival expectations, as found in previous literature in several other countries. Higher levels of education and income have a positive association with survival expectations, and these associations persist even when we condition on self-reported health. We find significant state level differences in survival expectations. The results remain robust to several alternative specifications.

We then compare the survival measure to objective measures of health. The distinct advantage of anthropometric and biomarker data is that they are objective markers of health and free from respondent reporting errors. We find that ADLs, height, and low hemoglobin levels covary with subjective expectations in expected directions. We also find that survival expectations are predictive of investments for the future. Overall, our findings suggest that researchers can ask subjective expectations of older survey respondents in a context such as India.

## Electronic supplementary material

ESM 1 (PDF 468 kb)

## Footnotes

1 Although other studies have elicited expectations in India (Delavande et al. 2011a; Giné et al. 2009; Tarozzi et al. 2014), this article presents the first results based on survival expectations from a representative sample of older Indians.

2 Using U.S. data, Hudomiet et al. (2015) found that asking respondents the probability of living at least X more years (with X being age- and gender-dependent and chosen in each group so that they correspond to similar life table probabilities), on average, reduces the age-related bias.

3 We also find similar qualitative results when using the fractional logit model for the relationship between objective measures of health and survival expectations presented in the sections, Activities of Daily Life and Biomarkers. The only exception is for the equivalent of Table 8, where low hemoglobin has a positive coefficient that is statistically significant at 10 % for women’s 1- and 5-year survival.

4 Recoding responses of 0 to 0.1 and responses of 1 to 0.9 does not alter our results significantly.

5 Savings is a subcomponent of an answer to the question, “Do you or members of your household possess any of the following financial assets? 1. Current accounts. 2. Savings accounts. 3. Stocks or mutual funds. 4. Bonds. 5. Outstanding balances in kitty parties, chit funds, bishi, and so forth. 6. Other. 7. None of the above.” Thus, our sample size is lower because it includes only the savings subcomponent of this response, with 0 indicating respondents who do not report any savings.

## References

[CR1] Aguila, E., Borges, A., Castillejos, C. M., Pierson, A., & Weidmer, B. A. (2014). *Mortality expectations of older Mexicans: Development and testing of survey measures *(Technical report). Santa Monica, CA: RAND.

[CR2] Arcidiacono P, Hotz VJ, Kang S (2012). Modeling college major choices using elicited measures of expectations and counterfactuals. Journal of Econometrics.

[CR3] Arokiasamy P, Bloom D, Lee J, Feeney K, Ozolins M, Smith AP, Majmundar M (2012). Longitudinal aging study in India: Vision, design, implementation, and preliminary findings. Aging in Asia: Findings from new and emerging data initiatives.

[CR4] Balia S (2014). Survival expectations, subjective health and smoking: Evidence from SHARE. Empirical Economics.

[CR5] Bhalotra S, Rawlings SB (2011). Intergenerational persistence in health in developing countries: The penalty of gender inequality?. Journal of Public Economics.

[CR6] Bloom, D., Hu, P., Arokiasamy, P., Risbud, A., Sekher, T. V., Mohanty, S. K., . . . Lee, J. (2014). *Longitudinal Aging Study in India: Biomarker documentation* (RAND Working Paper WR-1043). Santa Monica, CA: RAND.

[CR7] Bloom DE (2005). Education and public health: Mutual challenges worldwide. Comparative Education Review.

[CR8] Bloom, D. E., Canning, D., Moore, M., & Song, Y. (2006). *The effect of subjective survival probabilities on retirement and wealth in the United States* (NBER Working Paper No. 12688). Cambridge, MA: National Bureau of Economic Research.

[CR9] Burström B, Fredlund P (2001). Self rated health: Is it as good a predictor of subsequent mortality among adults in lower as well as in higher social classes?. Journal of Epidemiology and Community Health.

[CR10] Central Intelligence Agency (CIA) (2010). The world factbook.

[CR11] Chen, B., & Mahal, A. (2010). Measuring the health of the Indian elderly: Evidence from National Sample Survey data. *Population Health Metrics, 8,* 30. doi:10.1186/1478-7954-8-30 10.1186/1478-7954-8-30 PMC2993654 21080940

[CR12] de Bruin WB, Fischhoff B, Millstein SG, Halpern-Felsher BL (2000). Verbal and numerical expressions of probability: It’s a fifty–fifty chance. Organizational Behavior and Human Decision Processes.

[CR13] Delavande A (2008). Pill, patch, or shot? Subjective expectations and birth control choice. International Economic Review.

[CR14] Delavande A (2014). Probabilistic expectations in developing countries. Annual Review of Economics.

[CR15] Delavande, A., Giné, X., & McKenzie, D. (2011a). Eliciting probabilistic expectations with visual aids in developing countries: How sensitive are answers to variations in elicitation design? *Journal of Applied Econometrics, 26,* 479–497.

[CR16] Delavande, A., Giné, X., & McKenzie, D. (2011b). Measuring subjective expectations in developing countries: A critical review and new evidence. *Journal of Development Economics, 94,* 151–163.

[CR17] Delavande A, Kohler H-P (2009). Subjective expectations in the context of HIV/AIDS in Malawi. Demographic Research.

[CR18] Delavande A, Kohler H-P (2016). HIV/AIDS-related expectations and risky sexual behaviour in Malawi. Review of Economic Studies.

[CR19] Delavande, A., Perry, M., & Willis, R. (2006). *Probabilistic thinking and early Social Security claiming* (MRRC Working Paper No. 2006–129). Ann Arbor: Michigan Retirement Research Center.

[CR20] Delavande A, Rohwedder S (2011). Differential survival in Europe and the United States: Estimates based on subjective probabilities of survival. Demography.

[CR21] Delavande A, Rohwedder S (2011). Individuals’ uncertainty about future social security benefits and portfolio choice. Journal of Applied Econometrics.

[CR22] De Paula, A., Shapira, G., & Todd, P. E. (2014). How beliefs about HIV status affect risky behaviors: Evidence from Malawi. *Journal of Applied Econometrics, 29,* 944–964.

[CR23] Dormont, B., Samson, A.-L., Fleurbaey, M., Luchini, S., Schokkaert, E., Thébaut, C., & Van de Voorde, C. (2014). *Individual uncertainty on longevity* (KU Leuven Discussion Paper Series DPS14.28). Leuven, Belgium: KU Leuven.

[CR24] Elder, T. E. (2007). *Subjective survival probabilities in the Health and Retirement Study: Systematic biases and predictive validity *(MRRC Working Paper No. 2007–159). Ann Arbor: Michigan Retirement Research Center.

[CR25] Giné, X., Townsend, R., & Vickery, J. (2009). Forecasting when it matters: Evidence from semi-arid India. Unpublished manuscript, Federal Reserve Bank of New York, New York, NY.

[CR26] Groneck, M., Ludwig, A., & Zimper A. (2016). *The impact of biases in survival beliefs on savings behavior* (Working paper). Stockholm, Sweden: Stockholm School of Economics.

[CR27] Guralnik JM, Eisenstaedt RS, Ferrucci L, Klein HG, Woodman RC (2004). Prevalence of anemia in persons 65 years and older in the United States: Evidence for a high rate of unexplained anemia. Blood.

[CR28] Hamermesh DS (1985). Expectations, life expectancy, and economic behavior. Quarterly Journal of Economics.

[CR29] Hudomiet, P., Hurd, M., Kezdi, G., Rohwedder, S., & Willis R. J. (2015). Are the elderly overly optimistic about survival chances? Unpublished manuscript, Rand, Santa Monica, CA.

[CR30] Hudomiet, P., Hurd, M., & Rohwedder, S. (2016). Probability numeracy: Measurement and applications. Unpublished manuscript, Rand, Santa Monica, CA.

[CR31] Hurd MD (2009). Subjective probabilities in household surveys. Annual Review of Economics.

[CR32] Hurd MD, McGarry K (1995). Evaluation of the subjective probabilities of survival in the Health and Retirement Study. Journal of Human Resources.

[CR33] Hurd MD, McGarry K (2002). The predictive validity of subjective probabilities of survival. Economic Journal.

[CR34] Hurd MD, Smith JP, Zissimopoulos JM (2004). The effects of subjective survival on retirement and Social Security claiming. Journal of Applied Econometrics.

[CR35] Idler EL, Benyamini Y (1997). Self-rated health and mortality: A review of twenty-seven community studies. Journal of Health and Social Behavior.

[CR36] Kézdi, G., & Willis, R. J. (2011). *Household stock market beliefs and learning* (NBER Working Paper No. 17614). Cambridge, MA: National Bureau of Economic Research.

[CR37] Levin IP, Schneider SL, Gaeth GJ (1998). All frames are not created equal: A typology and critical analysis of framing effects. Organizational Behavior and Human Decision Processes.

[CR38] Lochner, L. (2007). Individual perceptions of the criminal justice system. *American Economic Review, 97,* 444–460.

[CR39] Manski CF (2004). Measuring expectations. Econometrica.

[CR40] Manski CF, Molinari F (2010). Rounding probabilistic expectations in surveys. Journal of Business & Economic Statistics.

[CR41] McKenzie D, de Mel S, Woodruff C (2008). Returns to capital: Results from a randomized experiment. Quarterly Journal of Economics.

[CR42] McKenzie D, Gibson J, Stillman S (2013). A land of milk and honey with streets paved with gold: Do emigrants have over-optimistic expectations about incomes abroad?. Journal of Development Economics.

[CR43] Menon, S. (2015). Out-of-range biomarker results and subjective survival expectations in ELSA. Unpublished manuscript, Department of Economics, European University Institute, Fiesole, Italy.

[CR44] Miller PM, Fagley NS (1991). The effects of framing, problem variations, and providing rationale on choice. Personality and Social Psychology Bulletin.

[CR45] Monden CWS, Smits J (2009). Maternal height and child mortality in 42 developing countries. American Journal of Human Biology.

[CR46] O’Broin SD, Gunter EW (1999). Screening of folate status with use of dried blood spots on filter paper. American Journal of Clinical Nutrition.

[CR47] Office of the Registrar General, India (2012). *Sample Registration System Statistical Report 2012* (Report No. 1 of 2013). New Delhi, India: Office of the Registrar General. Retrieved from http://www.censusindia.gov.in/vital_statistics/SRS_Based/India_2006-10.pdf

[CR48] Papke LE, Wooldridge JM (1996). Econometric methods for fractional response variables with an application to 401(k) plan participation rates. Journal of Applied Econometrics.

[CR49] Perozek M (2008). Using subjective expectations to forecast longevity: Do survey respondents know something we don’t know?. Demography.

[CR50] Schoenbaum M (1997). Do smokers understand the mortality effects of smoking? Evidence from the Health and Retirement Survey. American Journal of Public Health.

[CR51] Shapira, G. (2013). *How subjective beliefs about HIV infection affect life-cycle fertility: Evidence from rural Malawi* (World Bank Policy Research Working Paper No. 6343). Washington, DC: World Bank.

[CR52] Steckel RH (1979). Slave height profiles from coastwise manifests. Explorations in Economic History.

[CR53] Subramanian SV, Subramanyam MA, Selvaraj S, Kawachi I (2009). Are self-reports of health and morbidities in developing countries misleading? Evidence from India. Social Science & Medicine.

[CR54] Tarozzi A, Mahajan A, Blackburn B, Kopf D, Krishnan L, Yoong J (2014). Micro-loans, insecticide-treated bednets, and malaria: Evidence from a randomized controlled trial in Orissa, India. American Economic Review.

[CR55] Tolentino, K., & Friedman, J. F. (2007). An update on anemia in less developed countries. *American Journal of Tropical Medicine and Hygiene, 77,* 44–51. 17620629

[CR56] Tversky A, Kahneman D (1981). The framing of decisions and the psychology of choice. Science.

[CR57] United Nations. (2010). *World urbanization prospects, the 2009 revision: Highlights*. New York, NY: United Nations.

[CR58] Van der Klaauw W, Wolpin KI (2008). Social Security and the retirement and savings behavior of low-income households. Journal of Econometrics.

[CR59] Winter, J. (2008). Review of Robert Clark, Naohiro Ogawa, and Andrew Mason “Population aging, intergenerational transfers and the macroeconomy,” *Ageing and Society, 28,* 1049–1051.

[CR60] World Health Organization (WHO). (2001). *Iron deficiency anaemia: Assessment, prevention and control. A guide for programme managers*. Geneva, Switzerland: WHO.

[CR61] World Health Organization (WHO). (2011). *World health statistics*. Geneva, Switzerland: WHO.

[CR62] Zafar B (2013). College major choice and the gender gap. Journal of Human Resources.

