# Supplementary material for: Eliciting Survival Expectations of the Elderly in Low-Income Countries: Evidence From India
Source: Demography. 2017 Mar 9;54(2):673–99. doi: 10.1007/s13524-017-0560-8 (PMC5371617; doi:10.1007/s13524-017-0560-8)
Supplement: Supplementary file 1 — (PDF 468 kb) [file 13524_2017_560_MOESM1_ESM.pdf]

Eliciting Survival Expectations of the Elderly in Low-income Countries: Evidence from India

Online Resource 1

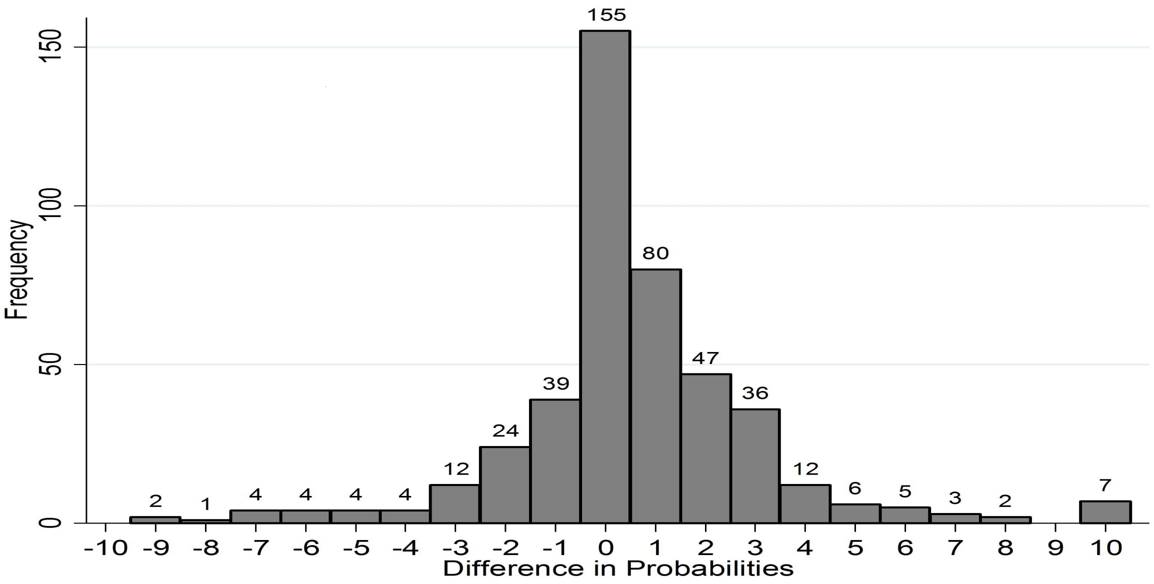

Figure S1: Difference in probabilities of going to the market

Table S1: Difference between own survival and hypothetical person survival

	Basic Reg			Baisc Reg + self health controls		
	**1-year survival**	**5-year survival**	**10-year survival**	**1-year survival**	**5-year survival**	**10-year survival**
Male						
Female	-0.012	0.051	0.048	-0.01	0.052	0.049
	[0.052]	[0.036]	[0.037]	[0.049]	[0.036]	[0.039]
45-54 years						
55-64 years	0.005	0.029	0.018	0.018	0.032	0.023
	[0.047]	[0.044]	[0.051]	[0.047]	[0.045]	[0.050]
65-74 years	0.035	0.019	-0.026	0.076	0.051	-0.022
	[0.063]	[0.063]	[0.061]	[0.068]	[0.065]	[0.064]
Over 75 years	0.068	-0.087	-0.019	0.139**	-0.05	-0.005
	[0.065]	[0.066]	[0.070]	[0.067]	[0.059]	[0.072]
No Schooling						
Primary/Middle School	-0.01	0.007	-0.003	-0.006	0.02	-0.003
	[0.061]	[0.051]	[0.054]	[0.056]	[0.052]	[0.053]
High School or more	0.059	0.062	0.048	0.052	0.046	0.055
	[0.087]	[0.079]	[0.067]	[0.082]	[0.077]	[0.067]
Other Caste						
Schedule Caste	-0.04	0.05	0.043	-0.044	0.041	0.039
	[0.084]	[0.081]	[0.073]	[0.069]	[0.081]	[0.072]
Schedule Tribe	-0.041	-0.021	0.023	-0.026	0.01	0.032
	[0.077]	[0.073]	[0.068]	[0.071]	[0.070]	[0.069]
Other Backward Caste	0.061	-0.019	-0.03	0.06	-0.029	-0.033
	[0.058]	[0.056]	[0.053]	[0.057]	[0.053]	[0.054]
Mortality Format						
Survival Format	-0.003	0.025	0.017	-0.018	0.021	0.012
	[0.044]	[0.033]	[0.039]	[0.043]	[0.031]	[0.037]
Both parents alive						
One or both parents are dead	-0.074	-0.024	-0.035	-0.109	-0.043	-0.028
	[0.094]	[0.087]	[0.076]	[0.094]	[0.074]	[0.070]
Income-Well below Average						
Income - Below Average	0.154**	-0.003	-0.021	0.174***	-0.002	-0.003
	[0.064]	[0.067]	[0.069]	[0.058]	[0.071]	[0.068]
Income - About Average	0.088*	-0.033	-0.089	0.111**	-0.035	-0.072
	[0.051]	[0.072]	[0.058]	[0.043]	[0.074]	[0.060]
Income - Well Off	-0.021	-0.033	-0.065	-0.033	-0.046	-0.06
	[0.064]	[0.093]	[0.077]	[0.057]	[0.094]	[0.077]
Punjab						
Rajasthan	-0.061	0.033	-0.073	-0.034	0.026	-0.074
	[0.069]	[0.066]	[0.053]	[0.070]	[0.065]	[0.058]
Kerala	0.023	0.055	-0.021	0.11	0.087	-0.022
	[0.074]	[0.055]	[0.063]	[0.076]	[0.073]	[0.070]
Karnataka	-0.058	-0.084	-0.132**	-0.028	-0.074	-0.122**
	[0.062]	[0.064]	[0.052]	[0.066]	[0.066]	[0.056]
Self Health-Very Good						
Self Health-Good				-0.510***	-0.11	-0.229**
				[0.061]	[0.127]	[0.093]
Self Health-Fair				-0.520***	-0.09	-0.219**
				[0.069]	[0.142]	[0.100]
Self Health-Poor				-0.743***	-0.336**	-0.248*
				[0.117]	[0.160]	[0.140]
Self Health-Very poor				-0.768***	-0.216	-0.055
				[0.187]	[0.176]	[0.126]
Cons	0.015	-0.004	0.092	0.518***	0.117	0.291**
	[0.102]	[0.143]	[0.116]	[0.090]	[0.154]	[0.140]
N	243	247	253	243	247	253

Regressions weighted by the pooled individual weights to provide survey design adjusted standard errors. Robust standard errors clustered at state level in parenthesis *** $p < 0.01$, ** $p < 0.05$, * $p < 0.1$.
All covariates are coded as binary indicators.

Table S2: Comparison of subjective survival expectations and state life table estimates by age group

Statistics	Overall		45-54 years		55-64 years		65-74 years		75+ years	
	**5-year survival**	**10-year survival**	**5-year survival**	**10-year survival**	**5-year survival**	**10-year survival**	**5-year survival**	**10-year survival**	**5-year survival**	**10-year survival**
**Panel A - Subjective Survival Expectations**										
Mean	0.61	0.55	0.62	0.57	0.64	0.58	0.59	0.49	0.51	0.48
p50	0.60	0.50	0.60	0.50	0.60	0.50	0.60	0.50	0.50	0.50
p25	0.40	0.30	0.40	0.40	0.40	0.30	0.40	0.30	0.40	0.30
p75	0.80	0.80	0.90	0.80	0.90	0.80	0.80	0.60	0.70	0.70
N	400	400	182	182	119	119	67	67	35	35
**Panel B - Life Table Estimates**										
Mean	0.84	0.68	0.95	0.87	0.86	0.69	0.73	0.43	0.35	0.08
p50	0.91	0.78	0.94	0.86	0.87	0.70	0.72	0.45	0.36	0.08
p25	0.79	0.56	0.93	0.83	0.84	0.63	0.7	0.32	0.24	0.04
p75	0.94	0.86	0.96	0.90	0.89	0.74	0.76	0.52	0.47	0.12
N	400	400	182	182	119	119	67	67	35	35
Unpaired t-test for equality of means*	0.00	0.00	0.00	0.00	0.00	0.00	0.00	0.10	0.00	0.00
